# The impact of fire suppression tasks on firefighter hydration: a critical review with consideration of the utility of reported hydration measures

**DOI:** 10.1186/s40557-016-0152-x

**Published:** 2016-11-15

**Authors:** Adam Walker, Rodney Pope, Robin Marc Orr

**Affiliations:** 1 Faculty of Health Sciences and Medicine, Bond Institute of Health and Sport, Bond University, Gold Coast, QLD 4226 Australia; 2 Tactical Research Unit, Bond University, Gold Coast, QLD 4226 Australia

**Keywords:** Dehydration, Firefighting, Structural fire, Wildland fire, Rural fire

## Abstract

**Background:**

Firefighting is a highly stressful occupation with unique physical challenges, apparel and environments that increase the potential for dehydration. Dehydration leaves the firefighter at risk of harm to their health, safety and performance. The purpose of this review was to critically analyse the current literature investigating the impact of fighting ‘live’ fires on firefighter hydration.

**Methods:**

A systematic search was performed of four electronic databases for relevant published studies investigating the impact of live fire suppression on firefighter hydration. Study eligibility was assessed using strict inclusion and exclusion criteria. The included studies were critically appraised using the Downs and Black protocol and graded according to the Kennelly grading system.

**Results:**

Ten studies met the eligibility criteria for this review. The average score for methodological quality was 55 %, ranging from 50 % (‘fair’ quality) to 61 % (‘good’ quality) with a ‘substantial agreement’ between raters (*k* = .772). Wildfire suppression was considered in five studies and structural fire suppression in five studies. Results varied across the studies, reflecting variations in outcome measures, hydration protocols and interventions. Three studies reported significant indicators of dehydration resulting from structural fire suppression, while two studies found mixed results, with some measures indicating dehydration and other measures an unchanged hydration status. Three studies found non-significant changes in hydration resulting from wildfire firefighting and two studies found significant improvements in markers of hydration. Ad libitum fluid intake was a common factor across the studies finding no, or less severe, dehydration.

**Conclusions:**

The evidence confirms that structural and wildfire firefighting can cause dehydration. Ad libitum drinking may be sufficient to maintain hydration in many wildfire environments but possibly not during intense, longer duration, hot structural fire operations. Future high quality research better quantifying the effects of these influences on the degree of dehydration is required to inform policies and procedures that ensure firefighter health and safety.

## Background

Firefighting is a highly stressful occupation, with firefighters exposed to a multitude of physical and environmental stresses during their normal duties [1]. Work can be highly variable, ranging from short duration structural fires, to long shifts (10–15 h) over multiple days (3–5 days) when fighting wildland fires [2]. In addition, this work can involve both low intensity exercise of long duration and periods of high intensity exercise of unpredictable duration [3–5]. Perhaps of most distinction, firefighting tasks are often performed in environmental extremes, such as high heat and dense smoke, while wearing impermeable, heavy and restrictive Personal Protective Equipment (PPE) [2, 6–8].

These factors create a uniquely arduous occupation, leading to significant heat and cardiovascular strain [1, 9–11]. Dehydration is one likely consequence [6]. This is due to the significant challenge to the thermoregulatory and body water balance systems caused by wearing PPE, the physical demands of firefighting and the high temperatures in which firefighters operate [1, 6, 12]. Firefighter PPE, which is typically heavy, thick and encompasses the head, induces profuse sweating [10]. The physical demands of firefighting combined with the substantially reduced water permeability, evaporative heat capacity and increased energy demands of wearing the PPE mean firefighter cooling capacity is reduced, causing the observed high sweat rates—in turn resulting in dehydration [10, 13–15].

Dehydration has been identified as having the potential to impact firefighter health, safety and performance [6, 16], and may exacerbate the effects of heat exposure by impairing the firefighter’s thermoregulatory response [17]. If firefighters are not receiving adequate fluid they may experience the adverse effects of dehydration [6, 18]. Dehydration is known to impair both cognitive and cardiovascular function [10] and reduces tolerance time in uncompensable conditions when compared to euhydration [19]. It should also be noted that *excessive* fluid intake during sustained physical activity can be equally dangerous, if it is sufficient to cause exercise-associated hyponatremia, or dangerously-low sodium concentrations in key body fluids [20]. As such, any hydration guidelines need to balance these concerns [21].

Accurately documenting the effects of firefighting on hydration status across multiple scenarios will enable the development or refinement of health and safety guidelines for firefighters [18]. Many fire agencies currently prescribe fluid intake through guidelines, with large variability (500–3000 mL/h), to combat occupational stresses [18]. Raines et al. (2015) state that these guidelines are largely based on data from sport and other occupations, due to the limited evidence in firefighting. Carlton and Orr [22], in a critical review on the effects on fluid loss on physical performance, state that the impacts of dehydration need to be studied in the specific environmental context.

On this basis, the purpose of this review was to critically appraise the current literature investigating the impact of fighting actual, or ‘live’, fires on firefighter hydration.

## Methods

### Search Strategy

A three layered search strategy was used to locate original articles for this review. Firstly, a comprehensive, systematic search of online literature indexing databases (PubMed, CINAHL, ProQuest and Google Scholar) was performed. Table 1 details the databases searched, and the search terms and filters used. Secondly, a manual search of the reference lists of articles retrieved in full text following screening and selection of identified studies was performed and results cross-checked against the initial database articles. Finally, to increase the field of potentially relevant articles and reduce the risk of publication bias affecting the results of the review, known researchers with a background in this field were approached and requested to provide further literature.

**Table 1:** Search strategy: Databases used, search terms, and filters applied

Database	Search terms	Filters
PubMed	(firefighter [Mesh] OR firefighter* OR “fire service” OR “fire fighter*” OR “fire and rescue personnel”) AND (hydration OR dehydration OR rehydration OR physiological* OR thermoregulation OR “thermal strain” OR temperature OR “water turnover” OR “fluid intake” OR drinking OR “fluid consumption”)	2000–2015
CINAHL	(firefighter [Mesh] OR firefighter* OR “fire service” OR “fire fighter*” OR “fire and rescue personnel”) AND (hydration OR dehydration OR rehydration OR physiological* OR thermoregulation OR “thermal strain” OR temperature OR “water turnover” OR “fluid intake” OR drinking OR “fluid consumption”)	2000–2015
ProQuest	(firefighter* OR “fire service” OR “fire fighter*” OR “fire and rescue personnel”) AND (hydration OR dehydration OR rehydration OR physiological* OR thermoregulation OR “thermal strain” OR temperature OR “water turnover” OR “fluid intake” OR drinking OR “fluid consumption”)	2000–2015
		Peer reviewed
		Terms in abstract
Google Scholar	(firefighter* OR fire service OR fire fighter* OR fire and rescue personnel) AND (hydration OR dehydration OR rehydration OR physiological response OR thermoregulation OR body temperature OR water turnover OR fluid intake OR drinking OR fluid consumption)	2000–2015

### Study Screening and Selection

**Fig. 1:**
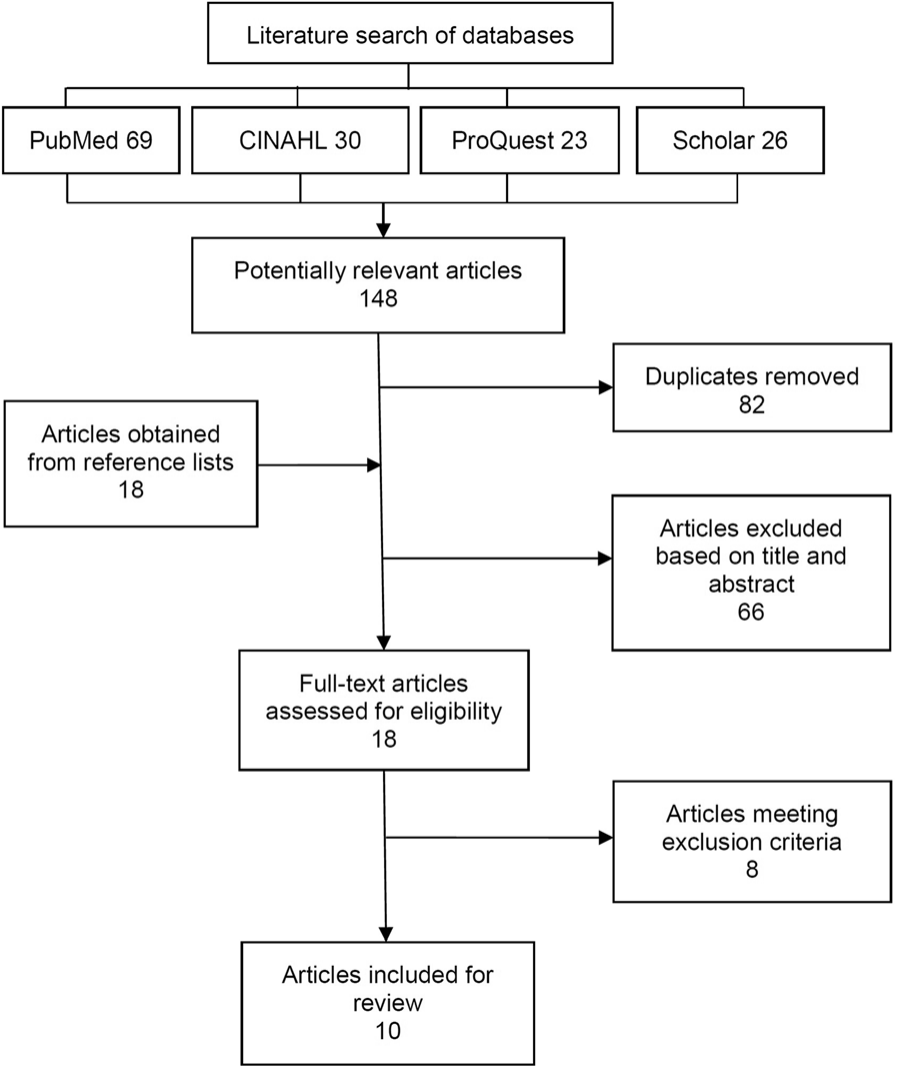
PRISMA flow chart of the literature screening and selection processes

Following retrieval of all potentially relevant articles, duplicates were removed and one reviewer screened the abstracts and titles against the inclusion criteria. Next, two reviewers met and reviewed the remaining articles in full text to assess their eligibility using the inclusion and exclusion criteria detailed below, until consensus was reached. A third author was available to mediate where consensus could not be reached, but was not required. A PRISMA flow diagram (Fig. 1; [23]) was developed to document the results of the literature search, screening and selection processes.

### Inclusion and exclusion criteria

For inclusion in this review, the articles had to meet specific, predetermined inclusion criteria. The inclusion criteria were: 1) the article specifically investigated the effect of fighting a ‘live’ fire on hydration; 2) firefighters were wearing full PPE; 3) the article was published in the last 15 years (due to changes in firefighter PPE); 4) the research involved human participants; 5) the article was published in English; and 6) the article was an original research article. Where possible, the inclusion criteria were applied through database filters (Table 1), but they were otherwise applied manually.

Following assessment of identified studies against the inclusion criteria, the remaining articles were assessed in full text against the exclusion criteria. The exclusion criteria were: 1) the article was a review, a poster, a case study or a thesis; 2) the research studied hydration during recovery from a live fire, rather than immediately following exposure; or 3) the article investigated the use of supplements.

### Critical Appraisal

The methodological quality of each included article was assessed using the Downs and Black protocol [24]. The Downs and Black protocol utilises a 27-question checklist to assess the quality of both randomised and non-randomised controlled studies and other types of observational studies. It is a suitable tool for use when conducting systematic reviews [25], and evaluates five key areas of methodological quality: reporting quality, external validity, internal validity—bias, internal validity—confounding, and statistical power [24]. The majority of the questions are scored no/unable to determine (=0 points) or yes (=1 point). Item 5, using a different scoring method, asks whether the authors have addressed potential confounding associated with baseline differences between groups in participant profiles and is scored from 0 to 2 points (0 points = no, 1 point = partially, and 2 points = yes). Item 27, normally scored on a 0–5 scale, was modified for the purposes of this review with 1 point awarded if a power or sample size calculation was reported and 0 points awarded if these calculations were absent; an approach employed in other critical reviews [26]. The total quality scores were converted into a percentage by dividing each article’s score by 28 and then multiplying that value by 100. They were then assigned one of three methodological quality ratings, as defined by Kennelly [27]: poor (<43 %), fair (43–57 %) or good (>57 %).

Each article was independently appraised and rated by two of the authors. The level of agreement between the two sets of ratings was measured using a Cohen’s Kappa analysis that considered all raw scores (27 scores per paper). Any disagreements in final scores were settled by the third author.

### Data extraction and synthesis

A systematic approach was used to extract key data from each included study. The key data included information on participants, outcome measures, interventions and main findings. One reviewer performed the data extraction from all included studies and the extracted data was cross-checked by a second reviewer.

Key data and findings from the included studies were synthesised and reported using a critical narrative approach. Themes for narration were based on the key outcome measures and interventions, while the synthesis took into consideration the methodological quality of each of the respective studies, and weighted the evidence from each accordingly.

## Results

A PRISMA flow diagram [23] showing the literature search, screening and selection results at each stage of the process is shown in Fig. 1.

A total of 148 potentially relevant articles were identified in the primary literature search (Fig. 1). The secondary search by hand of the reference lists of included articles yielded 18 further articles, while consulting subject matter experts did not produce any additional articles. A total of 10 articles examining the impact of fighting live fires on firefighter hydration were retained for critical appraisal and review [1, 2, 6–9, 12, 15, 18, 28].

### Critical appraisal of methodological quality

Table 2 lists the critical appraisal scores of the 10 included articles. The mean (±SD) Downs and Black [24] score for methodological quality of the 10 included studies was 56.43(±4.05)%, ranging from 50 [28] to 60.71 % [6, 7, 9], indicating a ‘fair’ to ‘good’ methodological quality of included studies, based on the Kennelly scale [27]. The kappa statistic for inter-rater agreement (*k* = .772, *p* < .001), indicated ‘substantial agreement’ between raters [29]. Good methodological quality [27] for *reporting* and *external validity* were found across the included studies, with mean (±SD) critical appraisal scores of 66.36 (±9.63) and 70 (±10.54), respectively, for these subscales of methodological quality. A lower score on the *internal validity*—*bias* subscale across the papers reflected limitations in the validity and reliability of outcome measures and the inability of authors to blind participants or researchers to the conditions in which participants operated. The inability of researchers to randomly allocate participants to intervention groups and the failure of authors to state *a priori* estimates of statistical power or required sample sizes based on power calculations led to lower scores for the *internal validity*—*confounding* and *statistical power* subscales, respectively.

**Table 2:** Key data, findings and critical appraisal scores (CAS) and methodological quality ratings of included articles

Study	Participants	Outcome measure(s)	Intervention(s)	Major Findings	CAS (%)
Cuddy et al. [1]	15 Hot Spot firefighters (12 male, 3 female) Wore PPE	Water turnover Nude BW	Live wildland fire suppression over 3 days of work shifts (11.4 ± 0.7 h/day) (hiking, line dragging, laying hose, chain sawing, clearing brush, lookout, and scouting) Ad libitum drinking 27.5 ± 3.2 °C mean ambient temperature	No significant change (*p* = 0.12) in morning BW across 3 days (pre 77.3 ± 8.3 kg, post 77.0 ± 8.9 kg) Mean water turnover 9.5 ± 1.7 L/d	54 (Fair)
Raines et al. [2]	32 firefighters (31 male,1 female) Ad libitum(AD) group (*n* = 17), pre-shift (PR) group (*n* = 15) PPE	Plasma Osm Urine colour USG	PR group consumed 500 mL fluid bolus pre shift and ad libitum remainder of shift 7 days (9.9 ± 2.2 h/day AD, 9.7 ± 2.3 h/day PR) of wildfire suppression (intense but intermittent work, using chainsaws, applying water, carrying, transit time and briefings) Mild to warm ambient temperatures (15.8–26.4 °C)	No difference between groups in total fluid intake (3.4 ± 1.6 L AD, 3.7 ± 2.9 L PR, *p* = 0.730) No significant differences between groups for any hydration marker (urine colour, *p* = 0.44; USG, *p* = 0.92; plasma Osm, *p* = 0.41) Significant decrease in both urine colour (*p* = 0.001) and USG (*p* = 0.01) across the shift, but still dehydrated by end, though less than at start Plasma Osm indicates firefighters arrived on shift dehydrated and finish dehydrated (*p* < 0.0001)	57 (Fair)
Horn et al. [6]	35 career, volunteer and industrial firefighters (31 male, 4 female) Wore PPE and SCBA	Nude BW Urine colour USG Urine Osm Salivary Osm	3 h live fire training exercise in specialised training building. 3–4 evolutions lasting 15–25 min, separated by 10–15 min (obtain water, advance hoses, extinguish fires, forcible entries, search and rescue and ventilation tasks) Encouraged ad libitum drinking (water and sport drink) Cool autumn day	Significant BW loss of 1.1 ± 0.8 kg (*p* < 0.001) and 1.9 ± 0.9 kg (2.2 % BW loss) adjusted for fluid intake and voiding Significant (*p* < 0.001) increase in salivary Osm (pre 78.0 ± 21.5 mOsm/kg, post 49.0 ± 15 mOsm/kg), reflective of dehydration No significant (*p* > 0.05) changes in Urine Osm (pre 768 ± 260 mOsm/kg, post 712 ± 251 mOsm/kg), urine colour (pre score 5.8 ± 1.5, post score 5.3 ± 1.5), or USG (pre 1.025 ± 0.009, post 1.024 ± 0.009)	61 (Good)
Holsworth et al. [7]	9 volunteer firefighters (8 males, 1 female) WorePPE and SCBA	HCT	2 × 30 min strenuous live structural fires No fluid intake	Significant (*p* = 0.0014) change in HCT (pre 43.7 ± 3.1 %, post 46.1 ± 2.3 %), indicating significant dehydration	61 (Good)
Smith et al. [8]	11 male professional firefighters WorePPE and SCBA (20.4 kg)	HCT	3 trials of 5 standardised firefighting drills in a training structure (carrying a hose up 4 flights, hoisting a hose, discharging a pump can, dragging a dummy, chopping a block of wood). 10 min rest between trials No fluid intake Unknown ambient temperature	Significant (*p* < 0.001) change in HCT (pre 43.2 ± 0.75 %, post 47.3 ± 0.75 %), indicating significant dehydration	57 (Fair)
Angerer et al. [9]	49 firefighter trainees (all male) Wore PPE and SCBA (24 kg)	Nude BW	30 min fire operation in a large simulation plant (ascending stairs, dragging hoses, fire suppression, dragging 80 kg dummy up and downstairs, crouched progression) No fluid intake Maximum 200 °C at 1.5 m above ground and 700 °C below the ceiling	BW significantly (*p* <0.001) reduced by mean 0.6 ± 0.2 kg	61 (Good)
Raines et al. [12]	34 firefighters (32 male, 2 female) AD group (*n* = 17) PR group (*n* = 17) Wore PPE	Plasma Osm Urine colour USG	PR group consumed 600mLwater and 600 mL sport drink per hour of shift One day (9.9 ± 2.2 h/day AD, 10.5 ± 2.2 h/day PR) of wildfire suppression (intense but intermittent work, using chainsaws, applying water, carrying, transit time and briefings) Mild to warm ambient temperature (15.8–26.4 °C)	PR group significantly higher total water intake (7.1 ± 3.1 L PR, 3.4 ± 1.6 L AD, *p* < 0.001) Both groups began work dehydrated according to USG (1.019 ± 0.007 AD, 1.016 ± 0.005 PR) PR post shift had significantly (*p* = 0.001) lower USG (1.004 ± 0.002) than AD (1.016 ± 0.008) Change from dehydrated to euhydrated for PR group only Significant (*p* < 0.01) change in urine colour for both groups (pre score 2.8 ± 0.4 AD, 2.6 ± 0.5 PR, post score 2.3 ± 0.8 AD, 1.4 ± 0.5 PR), but still dehydrated Plasma Osm no different between groups (*p* = 0.371), both arrived dehydrated and post shift both significantly reduced (*p* < 0.0001) to achieve similar euhydration	57 (Fair)
Ruby et al. [15]	14 wildland firefighters Wore PPE	Water turnover USG Urine Osm	5 day period of arduous fire suppression (hiking with load and fire line construction) Ad libitum drinking Unspecified ambient temperature	Water turnover 6.7 ± 4 L/day No significant change in USG (pre 1.016 ± 0.006, post 1.108 ± 0.006) No significant change in urine Osm (pre 562 ± 175 mOsm/L, post 629 ± 216 mOsm/L)	50 (Fair)
Raines et al. [18]	12 male wildfire firefighters Wore PPE	Plasma Osm	2 days of 12 h live fire prescribed operation (controlled fire application, building containment lines with hoe, chasing spot fires and applying water) Ad libitum drinking Hot ambient temperature (30.9 ± 3.6 °C day 1, 32.8 ± 5.7 °C day 2)	No significant (*p* = 0.73) change in plasma osmolality (day 1 292 ± 1 mOsm/L, day 2 289 ± 0.5 mOsm/L), indicating euhydration throughout	57 (Fair)
Eglin et al. [28]	14 firefighter instructors (all male) Wore PPE and SCBA (20.2 kg)	USG Nude BW (adjusted) Fluid deficit	30 hot fire (HF) exercises (33 ± 7.9 min, 74 ± 42 °C) 6 fire behaviour (FB) exercises (26.3 ± 5.5 min, 45 ± 12 °C) and 8 fire attack (FA) exercises (7.3 ± 2.6 min, 139 ± 48 °C) performed on same day with 2 h rest in-between Ad libitum drinking	Mean BW change 0.96 ± 0.41 kg/h, fluid deficit 0.62 ± 0.61 L HF exercises (0.79 ± 0.80 %/h) Mean BW change 1.59 ± 0.57 kg for FB and FA exercises Mild hypohydration at end of exercises with insufficient fluid intake to counteract body weight loss from sweating No significant (*p* > 0.05) change in USG (pre 1.019 ± 0.08, post 1.021 ± 0.009)	50 (Fair)

*CAS* critical appraisal score, *PPE* personal protective equipment, *SCBA* self-contained breathing apparatus, *HCT* haematocrit, *USG* urine specific gravity, *BW* body weight, *Osm* osmolality

### Study participants

The number of participants in each study (Table 2) ranged from 11 to 49 persons, consisting of males only [8, 18, 28] or predominantly males with one to four females [1, 2, 6, 7, 9, 12]. One study [15] did not specify the gender of participants. All participants were firefighters of various backgrounds and experience.

### Outcome measures

A total of eight hydration outcome measures were used (Table 2). Body mass, adjusted for fluid intake [1, 6, 9, 28] was used in 4 studies and urine specific gravity (USG) (>1.029 = hypohydrated) [2, 6, 12, 15, 28] in 5 studies. Urine colour compared against standard urine colour charts [30] was used in 3 studies [2, 6, 12]. Osmolality of urine (>700 mOsm/kg = dehydration), plasma (>290 mOsm/L = dehydrated) and saliva (>200 mOsm/kg = dehydrated), each determined through freezing point depression analysis, were used in 2 studies [6, 15], 3 studies [2, 12, 18] and 1 study [6], respectively. Holsworth et al. [7] and Smith et al. [8] used haematocrit as a marker of hydration through blood samples and Cuddy et al. [1] and Ruby et al. [15] calculated water turnover by examining differences between the isotopic enrichment of an oral dose of tracer water given pre-exposure and the isotopic enrichment observed to remain in post-exposure voided urine.

### Interventions

The firefighting interventions were clearly described in all but two studies [7, 15] which did not provide sufficient detail on the tasks performed, the protocol between exposures, shift duration and ambient temperature. Two types of firefighting interventions were evident; wildland firefighting [1, 2, 12, 15, 18] and simulated structural firefighting exercises [6–9, 28].

The wildfire firefighting interventions allowed ad libitum drinking [1, 15, 18] or used a prescribed drinking protocol [2, 12]. Firefighting was performed in mild [2, 12] to hot [18] ambient temperatures over 1 day [2, 12], 2 days [18], 3 days [1] or 5 days [1]. All firefighters involved in the wildland studies wore standard PPE with no actual load weights described.

Structural fire scenarios were performed in specialised training facilities and consisted of a 30 min operation [9, 28], 2 × 30 min operations [7] or a 3 h operation [6]. The intervention used by Smith et al. [8] involved 5 standardised trials completed 3 times, each trial of an unspecified duration. Horn et al. [6] and Eglin et al. [28] allowed and encouraged ad libitum drinking during structural firefighting, while the remaining studies did not [7–9]. Ambient temperatures within the structural fire scenarios were reported, but inconsistently, and ranged from a low mean temperature of 45 °C [28] to a high of 200 °C [9]. Temperatures were not specified in the other 3 studies [6–8]. All firefighters in the structural fire suppression studies wore PPE with a self-contained breathing apparatus (SCBA) weighing 20.2 kg [28], 20.4 kg [8] or 24 kg [9]. PPE and SCBA weight was not stated in 2 studies [6, 7].

### Hydration outcomes

Table 2 summarises the main findings of the 10 studies, in relation to changes in firefighter hydration. Outcomes varied depending on the outcome measure and intervention.

Following structural fire interventions, the 3 studies [7–9] allowing no fluid intake all found a significant change in outcome measures, indicating dehydration. Horn et al. [6] and Eglin et al. [28] both allowed ad libitum drinking and found mixed results, with body weight (*p* < 0.001) and salivary osmolality (*p* < 0.001) indicating dehydration while USG, urine osmolality and urine colour (*p* > 0.05) indicated an unchanged hydration status.

Three studies found non-significant changes in hydration measures following wildfire firefighting [1, 15, 18]. Significant improvements or little change in markers of hydration were found following wildfire firefighting in the studies of Raines et al. [2, 12], indicating a change from dehydration at the start of the shift to euhydration or an unchanged hydration status by the end of shift.

## Discussion

Firefighting is a highly stressful occupation involving unique physical challenges, PPE and environments that increase the potential for dehydration to occur. The results of the studies included in this review indicate that dehydration is a reality in many firefighting contexts, but that this can be adequately addressed in most instances by ad libitum fluid intake. Dehydration leaves the firefighter at risk of harm to their health, safety and performance [6, 16]. The evidence provided by these studies assists in determining fluid replacement requirements for firefighters to combat occupational fluid loss. In general, the results of this review have shown fair to good quality evidence that firefighting will result in dehydration if adequate fluid is not provided or available to firefighters, over both short and long duration fire operations, but that if adequate fluid is available and ad libitum fluid intake is allowed and feasible, dehydration can be prevented, minimised or reduced in both structural and wildfire firefighting scenarios.

When considering the methodological quality of the included studies, there were some consistent weaknesses identified when viewed through the lens of the Downs and Black protocol, suggesting caution should be applied in interpretation of data. The lack of randomisation and blinding in the study designs, relatively small sample sizes (*n* = 11–49), and variability in interventions and outcome measures must all be considered when interpreting the findings. As must failures to state details of the firefighting interventions, such as ambient temperatures and durations of exposure. When comparing the studies involving similar interventions, preliminary conclusions can, nevertheless, be drawn, while taking into consideration the outcome measures used and their validity.

Results from those studies which utilised nude body weight [1, 6, 9, 28] demonstrated varying results. Previous research has shown that acute changes in body mass reflect changes in body water [31, 32]. A loss of greater than 1–2 % of body mass indicates insufficient fluid intake [33]. Both Angerer et al. [9] and Eglin et al. [28] demonstrated loss of body weight from 30 mins of structural firefighting of only 0.47 and 0.79 %/h, indicating hydration was maintained, with no drinking and ad libitum drinking, respectively. Horn et al. [6], on the other hand, found a mean body weight loss of 2.2 % over 3 h of structural firefighting with encouraged ad libitum drinking, indicating dehydration. Cuddy et al. [1] was the only author to study body weight changes in wildland fires and found no significant difference in body weight across 3 days of wild firefighting with ad libitum drinking. Studying body mass over multiple days introduces many sources of error, as weight can fluctuate for a variety of reasons including energy balance and glycogen stores [15], however it remains likely that hydration was well maintained in this group.

Urine-based hydration measures including USG, colour and osmolality were used throughout the selected studies, producing varying results. Horn et al. [6] and Eglin et al. [28] both found no significant change in urine measures following structural firefighting with ad libitum drinking. These results are in direct opposition to the reported significant loss of body mass in the study by Horn et al. [6]. Urine measure results followed similar trends in indicating hydration maintenance during wildland fire operations. Raines et al. [2, 12] found significant improvements in urine measures, whereby pre-intervention levels of hydration were maintained [2, 12] or a change from a dehydrated to an euhydrated state with higher water intake in the prescribed drinking group [12]. In the study by Ruby et al. [15], urine results showed no significant changes over 5 days of wildfire fighting with ad libitum drinking. Considering these findings, it should be noted that Horn et al. [6] were the only authors to state the timing of urine samples (within 30 min of intervention), with the potential range of timings being a factor that might affect the reliability of these measures, as USG and urine osmolality may lag behind during periods of rapid body fluid turnover due to the protective role of the kidneys [34].

Although urinary measures are associated with well-established indexes of dehydration [6], all authors expressed concerns regarding the validity of urinary measures and their poor correlation with other hydration measures, including body weight, total body water, and plasma and salivary osmolality [6, 15, 18]. Raines et al. [12] highlighted articles [34–36] indicating that there is little to no evidence that USG is sensitive to changes in hydration status, unlike plasma osmolality. As urine osmolality can be used interchangeably with USG, this also casts doubt on its clinical utility [37]. Authors suggested that a shift towards the use of plasma osmolality [2, 12] and salivary osmolality [6] as more valid measures of hydration was needed for clinical trials to improve methodological quality.

Plasma osmolality has been promoted as the current gold standard marker of hydration status [38]. A change of 5 to 13 mOsm/L signals an 80 % (likely) and 99 % (near certain) likelihood that a meaningful change in hydration status has occurred [39]. Considering this, Raines et al. [18] found no significant change (*p* = 0.73) in plasma osmolality following firefighting with ad libitum drinking, while in their earlier studies, Raines et al. [2, 12] found significant reductions (*p* < 0.0001) in plasma osmolality and a shift from dehydration at the start of a firefighter’s shift to euhydration by the end. These results differed from urine measure results but followed similar trends, indicating maintenance or improvement in hydration when ad libitum or prescribed drinking was implemented during wildland fire suppression.

The remaining measures used to determine hydration levels following structural fires consistently indicated dehydration following firefighting. Salivary osmolality has been shown to be a marker of acute hydration but questions still remain over its utility as a field measure due to concerns with the practicality of sample collection [40]. Salivary osmolality in response to a 3 h structural fire suppression exercise was investigated by Horn et al. [6]. Results were interpreted based on estimates of change in hydration and compared against body weight changes, as there were no salivary osmolality criterion measures of dehydration status [6]. Horn et al. [6] concluded that levels of dehydration increased post-fire exposure when drinking ad libitum was allowed and that with future research and the development of a suitable portable tool, salivary osmolality could be a reliable and valid field measure, overcoming the impracticality of nude body mass and urinary measures.

Holsworth et al. [7] states that haematocrit is an important blood marker of hydration. Both Holsworth et al. [7] and Smith et al. [8] found significant changes in haematocrit indicating dehydration, following structural firefighting without fluid intake. However, concerns regarding the differentiation between dehydration and heat stress were noted by Smith et al. [8], as changes in blood chemistry are typically transient and depend on the extent of hypohydration and cellular damage. The authors concluded that pre-hydration and rehydration need to be a priority in hot and arduous conditions.

Water turnover provides a valuable guide to drinking requirements needed to maintain hydration during wildland fires. A day of wildland fighting requires a minimum of 6–8 l to be ingested according to the results reported by Ruby et al. [15] or 8–11 l according to the results of Cuddy et al. [1], although Cuddy et al. [1] note that these values can vary considerably based on ambient temperature and drinking habits of each individual. Of note, both authors concluded that firefighters failed to consume sufficient fluids to maintain hydration. These fluid requirements are considerably greater than those reported by Raines et al. [18], who found intakes of 420 ± 132 ml/h in hot environments and 264 mL/h in mild to warm conditions, over 10 and 12 h shifts, were required.

Despite variations in outcome measures, results of the included studies of wildfire suppression all followed similar trends, indicating that ad libitum drinking was sufficient to maintain or improve hydration status across single or multiple day wildland fire suppression operations in cool to hot conditions [1, 2, 12, 15, 18]. Evidence in structural fires is less consistent, and utilised less valid measures of hydration. Results tended to indicate that regardless of whether ad libitum drinking is allowed or not, structural firefighting of greater than 1 h and possibly as short as 30 min resulted in some level of dehydration [6–9, 28]. This is likely due to the more intense physical nature, carriage of potentially heavier loads in PPE, higher environmental temperatures and limited opportunity to consume fluids during structural fire suppression, and extra effort may be required to ensure ready access to palatable fluid and opportunity to drink.

### Strengths and limitations of the critical review

Key strengths of this review are its systematic and critical approach and inclusion of 10 studies involving a variety of relevant firefighting scenarios, hydration measures and fluid consumption models. These factors have allowed for useful comparisons and consideration of possible reasons for observed heterogeneity in reported results, and these will usefully inform both future research and interim policies and procedures. The review was limited, however, by the relatively small number of studies meeting the review criteria and the methodological quality of these studies. Firm conclusions are difficult to draw, given these limitations and both the variable nature of the reported firefighting operations and the variation in reporting of tasks performed, time of exposure and ambient temperatures. Furthermore, the variance and questionable validity of some outcome measures limits the development of dedicated recommendations.

### Practical implications

The significance of quantifying dehydration that occurs during fighting of actual fires is recognised by fire agencies, with the release over previous years of guidelines targeted at minimising the risk of dehydration [41–44]. A better understanding of the incidence of dehydration assists in the development of such policies and procedures to ensure firefighter health and safety. Horn et al. (2012) state that guidelines from athletic populations provide a reasonable benchmark on which to base guidelines, but may have limited utility with firefighters due to PPE worn and higher ambient temperatures. It is clear that firefighting will result in dehydration if adequate fluids are not consumed. Further research into the fluid requirements to maintain hydration, particular with use of valid and reliable outcome measures such as plasma osmolality will increase the homogeneity of the evidence and provide a clearer understanding. Raines et al. [2, 12, 18] have provided good evidence quantifying the hydration requirements of fighting wildland fires in mild to hot conditions. However, due to the intense nature of structural firefighting and the potential for rapid dehydration, more research to determine fluid replacement requirements is particularly warranted. Future studies should report ambient temperatures, duration of exposure and justifications of outcome measures used. With further research, evidence based guidelines can be developed to ensure the health and safety of firefighters across multiple scenarios and environmental conditions. In the interim, general advice to firefighters and their managers to ensure ready access to fluids and to consume fluids ad libitum, guided primarily by thirst, would seem most appropriate and supported by the available evidence from this review and from previous research [21]. Care should be taken to avoid promoting *over*-*hydration*, as noted in the introduction to this review [21].

## Conclusion

In conclusion, there is fair to good evidence to indicate that firefighting results in dehydration when fluid intake is inadequate. This situation is exacerbated during intense activity and extreme environments. During wildfire operations, ad libitum drinking is sufficient to maintain hydration, however during structural fire suppression tasks, ad libitum drinking may not be sufficient to maintain hydration and dehydration can occur rapidly, possibly due to the intensity of the tasks and lack of time or ready access to fluids. As such, while ad libitum drinking should be encouraged during all firefighting tasks, special consideration may need to be given to ways to enhance access to fluids and capacity for ad libitum fluid intake while fighting structural fires, and this concern also warrants further investigation.

## Abbreviations

PPE: Personal protective equipment
SCBA: Self-contained breathing apparatus
USG: Urine specific gravity
